# Differences in functional niche hypervolume among four types of forest vegetation and their environmental determinants across various climatic regions in China

**DOI:** 10.3389/fpls.2023.1243209

**Published:** 2023-12-05

**Authors:** Jihong Huang, Ruoyun Yu, Runguo Zang

**Affiliations:** ^1^Key Laboratory of Forest Ecology and Environment of National Forestry and Grassland Administration, Ecology and Nature Conservation Institute, Chinese Academy of Forestry, Beijing, China; ^2^Co-Innovation Center for Sustainable Forestry in Southern China, Nanjing Forestry University, Nanjing, China; ^3^Coconut Research Institute, Chinese Academy of Tropical Agricultural Sciences, Wenchang, Hainan, China

**Keywords:** functional traits, functional niche hypervolumes, forest vegetation, environmental factors, ecological niche

## Abstract

Functional traits play an important role in studying the functional niche in plant communities. However, it remains unclear whether the functional niches of typical forest plant communities in different climatic regions based on functional traits are consistent. Here, we present data for 215 woody species, encompassing 11 functional traits related to three fundamental niche dimensions (leaf economy, mechanical support, and reproductive phenology). These data were collected from forests across four climatic zones in China (tropical, subtropical, warm-temperate, and cold-temperate) or sourced from the literature. We calculated the functional niche hypervolume, representing the range of changes in the multidimensional functional niche. This metric quantifies how many functional niche spaces are occupied by existing plants in the community. Subsequently, we analyzed differences in functional niche hypervolume and their associated environmental factors across different types of forest vegetation. The results indicate that the functional niche hypervolume and the degree of forest vegetation overlap decrease with increasing latitude (e.g., from tropical rainforest to cold temperate coniferous forest). The total explanatory power of both climate and soil factors on the variation in functional niche hypervolume was 50%, with climate factors exhibiting a higher explanatory power than soil factors. Functional niche hypervolume is positively correlated with climate factors (annual mean temperature and annual precipitation) and negatively correlated with soil factors (soil pH, soil organic matter content, soil total nitrogen content, and soil total phosphorus content). Among these factors, annual mean temperature, soil pH, and soil total nitrogen content most significantly affect the difference in functional niche hypervolume among forest vegetation. Our study emphasizes the significant variation in the functional niche hypervolume among typical forest vegetation in China.

## 1 Introduction

Functional niche reflects the adaptive strategies of plants in different environments, playing a significant role in studying mechanisms of community coexistence and predicting the species’ distribution along environmental gradient (Maynard et al., 2015; Jiang et al., 2018). Functional traits, which are crucial characteristics that permeate individuals, populations, communities, and ecosystems (Zhang et al., 2016), provide a new entry point for understanding ecosystem functions and processes. Functional niches, based on plant functional traits, have proven successful in predicting the roles of plant species in ecosystems (Diaz and Cabido, 2001; McGill et al., 2006; Lavorel et al., 2011). Research on functional niches, grounded in plant functional traits, has garnered increased attention in recent decades. The plant functional niche can be calculated by determining the functional position of species in the community through the average trait value of species and calculating the niche width as the difference in intraspecific traits (Violle and Jiang, 2009). Ecologists define functional niche as the relative position of species on the functional axis within the multidimensional niche space constructed based on the functional traits of species (Blonder, 2018; Li et al., 2018; Raffard et al., 2020).

Existing research demonstrates that a species’ niche exhibits obvious functional differentiation within the multidimensional functional niche space. Changes in the dominant functional dimension dictate the key ecological strategy employed by a species in response to its environment (Pianka et al., 2017). Structural differences in morphology between species can facilitate the separation of functional niches and balance competition among them. For instance, deciduous and evergreen plants occupy distinct functional niche spaces, with deciduous plants typically associated with higher soil nutrient demand and lower stress resistance (de la Riva et al., 2017). The functional niche of a species becomes more diverse as community species’ richness increases, leading to an expansion of the niche space accordingly (Li et al., 2018). Previous research indicates that environmental filtering is the primary factor driving changes in the functional niche, and a species’ functional niche maintains a covariant relationship with environmental changes (Yang et al., 2014). Significant differences in functional niche overlap were observed for herbaceous plants along the gradient of environmental interference and stress, particularly related to soil fertility (Li and Shipley, 2019). The degree of niche overlap is directly proportional to interference intensity in medium and low-stress environments.

To analyze the geographic difference in niche, it is essential to quantify the functional niche space and the degree of functional trait overlap within it. The proposed creation of a functional niche periodic table based on functional traits (Pianka et al., 2017) provides a practical foundation for quantitative analysis. The key to constructing such a niche periodic table is the scientific selection of different functional dimensions and the accurate placement of species on the axis of multidimensional niche space. An organism’s habitat, life history, nutrition, defense, and metabolism represent the most pertinent ecological strategies. Functional traits can be chosen based on these five dimensions to construct the niche periodic table (Winemiller et al., 2015). The niche periodic table has been successfully employed in studying the functional ecology of lizards (Pianka et al., 2017). However, the functional dimension of plants differs from that of animals, and it can be categorized into different groups based on how plant functional traits respond to the environment. This involves grouping traits that contain distinct information about functional dimensions (Kraft et al., 2015; Wang et al., 2018).

Environmental conditions represent the primary abiotic factors influencing the functional niche of plants, as environmental change serves as a major external driving force that maximizes plant functions. In essence, plants optimize their utilization of environmental resources by leveraging their strengths and mitigating their weaknesses (Gratani et al., 2003). Differences in the functional niches of species, reflecting various resource acquisition strategies among plants, may alleviate species competition in resource-rich environments. On a regional scale, environmental factors, particularly climate parameters such as average temperature and precipitation, exhibit certain regular changes across geographical gradients, thereby shaping the functional characteristics of plants through environmental filtering. Environmental filtering is considered to play a more significant role in temperate forests compared to tropical rainforests (Myers et al., 2013; Yao et al., 2020). At smaller scales, such as local plots, soil and terrain factors often come into play (Stein et al., 2014; Takahashi and Tanaka, 2016). Higher environmental pressure compels species to maintain a stronger functional balance (He et al., 2021).

Different environmental factors in various climatic zones drive the development of distinct functional traits in plants, resulting in diverse adaptive strategies (Westoby et al., 2002). The survival strategies of plants can be represented as their positions in the three-dimensional space of the leaf-height-seed (LHS) scheme (Westoby, 1998; Pollock et al., 2018; Fyllas et al., 2020). Species with similar functional traits are typically confined to a specific suitable area due to environmental filtering, leading to the occupation of distinct functional niche spaces by different species (Ackerly, 2003). Climatic conditions generally constrain the relationships between traits on a global scale (Butler et al., 2017; Šímová et al., 2018). The similarity of traits along climate gradients has been observed at global scales (Díaz et al., 2016; Wright et al., 2017). A forest endowed with abundant water and heat resources possesses a larger functional niche space than one with a less favorable environment (Yu, 2021). In contrast, the influence of soil is more evident at a small scale. Soil nutrients significantly impact the spatial variation of plant functional traits (Ordoñez et al., 2009). When soil nutrient content is low, plants tend to exhibit a higher degree of functional trait integration, which hinders the expansion of plant functional space (He et al., 2021).

While the quantity of research on plant functional traits has increased in recent years, there remains a noticeable gap in the study of plant functional niches and their responses to various environmental factors such as temperature, precipitation, and soil nutrient content (Zhang et al., 2020). By integrating the multidimensional functional traits of plants, plant functional niche more comprehensively reflects the overall trade-off strategies employed by plants. The functional niche hypervolume, representing the range of changes in the multidimensional functional niche (the volume of functional niche space), is utilized to quantify how many functional niche spaces are occupied by existing plants in the community (Blonder et al., 2014). To date, little is known about how the spatial differentiation of plant functional niche hypervolume responds to large-scale patterns of environmental change based on functional traits. This study focuses on woody plants in four forest types across different climatic regions in China: tropical rainforest (TF), subtropical evergreen deciduous broad-leaved mixed forest (SF), warm-temperate coniferous broad-leaved mixed forest (WF), and cold temperate coniferous forest (CF). The study aims to address the following questions: (1) Is there a difference in the functional niches’ hypervolume and overlap degree among different forest vegetation types? (2) What are the main factors affecting the difference in the functional niches’ hypervolume and degree of overlap in different forest vegetation types?

## 2 Materials and methods

### 2.1 The study area

**Figure 1:**
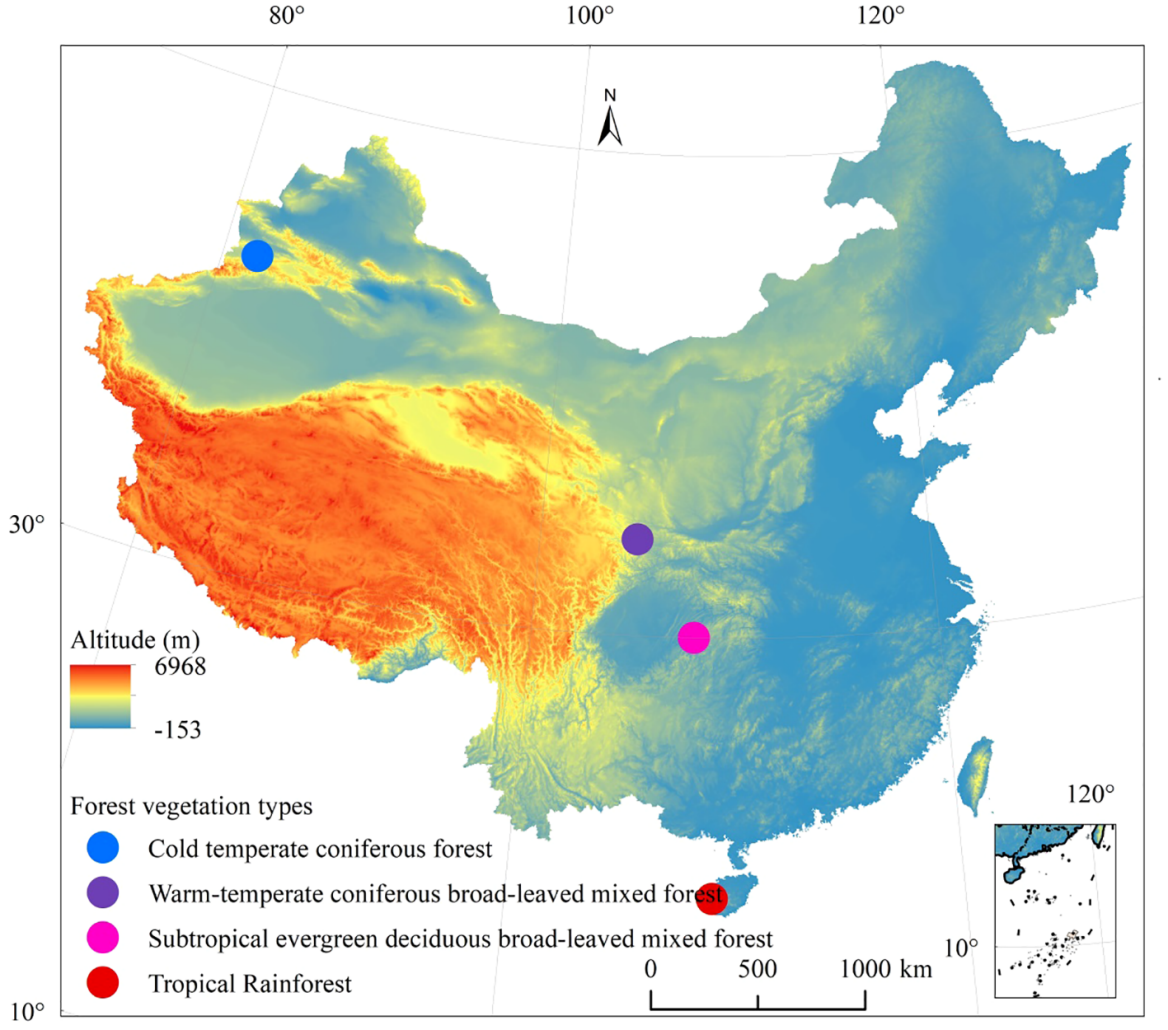
Distribution of four forest vegetation types in different climatic zones in China.

The study encompasses four distinct forest types, each located in specific regions: the TF area is situated in the Bawangling National Nature Reserve in Hainan Province; the SF area includes Mulinzi and Xingdoushan National Nature Reserve in Hubei Province; the WF area is found in the Xiaolongshan National Nature Reserve in Gansu Province; and the CF area is situated in the Kanas National Nature Reserve in Xinjiang Uygur Autonomous Region (Autonomous region is equivalent to province). The Bawangling TF represents one of the most well-preserved primitive tropical rainforests in China, with the Bawangling tropical mountain rainforest being the largest among the forest types in the reserve. The SF, located in Mulinzi, Hubei Province, particularly in the Xingdoushan forest area, exhibits distinct seasonal variations. The first forest layer mainly consists of deciduous broad-leaved trees, while the second is dominated by evergreen broad-leaved trees. The Xiaolongshan boasts unique geographical and environmental conditions, situated at the intersection of four natural vegetation regions (Himalaya, Mongolia Xinjiang Plateau, central China, and North China). Positioned at the south edge of the temperate zone, it exhibits a warm and temperate character. The geographical distribution of the vegetation is illustrated in **Figure 1**.

### 2.2 Sampling design

From April 2018 to July 2019, we randomly established 50 permanent 20 m×20 m forest dynamic plots (FDPs) in natural old-growth forests at each of the four study sites following the Center for Tropical Forest Science (CTFS) protocols (Condit, 1998). Within each plot, data for all woody plants (including trees and shrubs) with a diameter at breast height (DBH) ≥ 1 cm were recorded, including species name, DBH, location (coordinate within the plot), height, and growth status. All species were identified with the assistance of local botanists. In each vegetation type, at least 10 robust individual plants per species were sampled to measure their functional traits. Only species with an abundance of >20 individuals per plot were sampled, covering over 90% of all individuals in each of the 50 FDPs. A total of 215 species (belonging to 116 genera and 55 families) were sampled across the four biomes. For each sampled individual, we collected five fully expanded and healthy leaves and one 1-2 cm diameter branch for trait measurements.

Soil samples were also collected. Each of the 20m × 20m plots was divided into four 10m × 10m subplots. Soil samples (0–20 cm depth) were taken at the center of each subplot and the center of each 20m × 20m plot, resulting in a total of five samples collected and mixed for each FDP.

### 2.3 Functional traits

The functional niche space of woody plants was constructed by selecting 11 functional traits across three functional dimensions (Yu, 2021): (1) leaf economic traits, including leaf area (LA, cm^2^), specific leaf area (SLA, cm^2^/g), leaf dry matter content (LDMC, g/g), and leaf nitrogen concentration (LNC, g/cm^3^); (2) mechanical support characters, including stem tissue density (STD, g/cm^3^), potential maximum plant height (MPH, m), and potential maximum diameter at breast height (MDBH, CM); and (3) reproductive phenological traits, including mean flowering time (FLT, day), flowering duration (FLD, day), mean fruiting time (FRT, day), and fruiting duration (FRD, day). All 11 functional traits were collected from field sampling or identified in the literature. The seven functional traits in the economic and mechanical support dimensions of plant leaves were obtained through field sampling and their measurement adhered to standard methods outlined in the handbook for standardized measurement of plant functional traits (Pérez-Harguindeguy et al., 2013). LA was measured using the LI-COR3100C area meter (LI-COR, Lincoln, NE, United States). The leaf fresh mass was weighed and then dried for 72 hours in an oven at 60°C to obtain leaf dry mass. LDMC was calculated as the ratio of leaf dry mass to fresh mass, and SLA was calculated as the ratio of LA to leaf dry mass. LNC was measured by Kjeldahl digestion, followed by colorimetric analysis. To measure STD, one 5 cm long segment was cut from branches with a diameter between 1 and 2 cm. The pith, phloem, and bark from all segments were removed, and the segments’ fresh weight was measured using a Mettler-Toledo balance. The dry mass was then measured after drying for 72 h at 70°C. STD was calculated as the dry mass divided by fresh volume calculated from the entire sectional area (Pérez-Harguindeguy et al., 2013). MPH and MDBH were calculated as the 95th percentile value for each species based on at least 20 individuals across 50 FDPs in each study site (Fauset et al., 2015). The four functional traits in the reproductive phenology dimension were primarily collected from the flora of China (http://www.efloras.org/). FLT, FLD, FRT, and FRD were calculated using the Julian date (Laughlin et al., 2010).

### 2.4 Environmental variables

Climatic and soil factors data were collected for each FDP. Climatic variables comprised 19 bioclimatic variables (Hijmans et al., 2005) obtained from the WorldClim database (https://www.worldclim.org/). In this study, the Pearson correlation test was employed for screening, and a standard correlation coefficient exceeding 0.95 was used to eliminate climate factors exhibiting high spatial autocorrelation. Subsequently, the annual average temperature and annual precipitation variables were selected for subsequent data analysis. Soil factor data from the 20m × 20m quadrats were measured *in situ*. The topsoil from 0 to 20 cm was collected along the diagonal and center of each quadrat and mixed thoroughly. The soil samples were then stored in a dry location in the laboratory for natural air drying prior to analysis. Soil analyses included tests for pH, soil organic matter (SOM), total nitrogen (TN), total phosphorus (TP), available nitrogen (AN), available phosphorus (AP), and available potassium (AK).

### 2.5 Data analysis

Before data analysis, the Z-values of environmental data were standardized to eliminate dimensional differences between environmental factors. The “PCA of PCA” method was employed to derive the functional niche values (species load values) of species on the PC1, PC2, and PC3 axes from the functional niche spaces of woody plants within forest vegetation across different climatic regions. Subsequently, the n-dimensional hypervolume method (Blonder et al., 2014) was utilized to calculate the functional niche values of each 20m ×20m quadrat. The influence of environmental factors on the functional niche hypervolume of woody forest plants was then analyzed for different climatic regions. The explanatory power of climate and soil factors in differentiating functional niche hypervolume was evaluated using the variance partitioning method (Peres-Neto et al., 2006). The Pearson correlation test was employed to assess the correlation between the functional niche hypervolume and each environmental factor. Regression analysis, including variance partitioning, was used to further evaluate changes over time. Finally, the model selection and multi-model inference (MSMI) method were applied to construct the optimal model and test the significance of woody plant functional niche hypervolume of forest vegetation and its environmental factors across different climate regions (Calcagno and de Mazancourt, 2010). The MSMI method utilizes the AIC value as the standard for calculating the optimal model. The influence of environmental factors on the functional niche hypervolume of woody plants can be evaluated from the optimal model’s variable importance results, enabling the determination of the key environmental factors affecting differences in the functional niche hypervolume of woody plant communities across different climatic regions. The data analysis and plotting described above were performed using the R 3.6.0 program (R Core Team, 2019). PCA analysis utilized the “vegan” package, n-dimensional space hypervolume calculation used the “hypervolume” package, the MSMI method employed the “glmulti” and “mumin” packages, and correlation, regression analysis, and mapping used the “stats” package and “ggplot2” packages.

## 3 Results

### 3.1 Differences in the functional niche hypervolumes of forest vegetation

**Figure 2:**
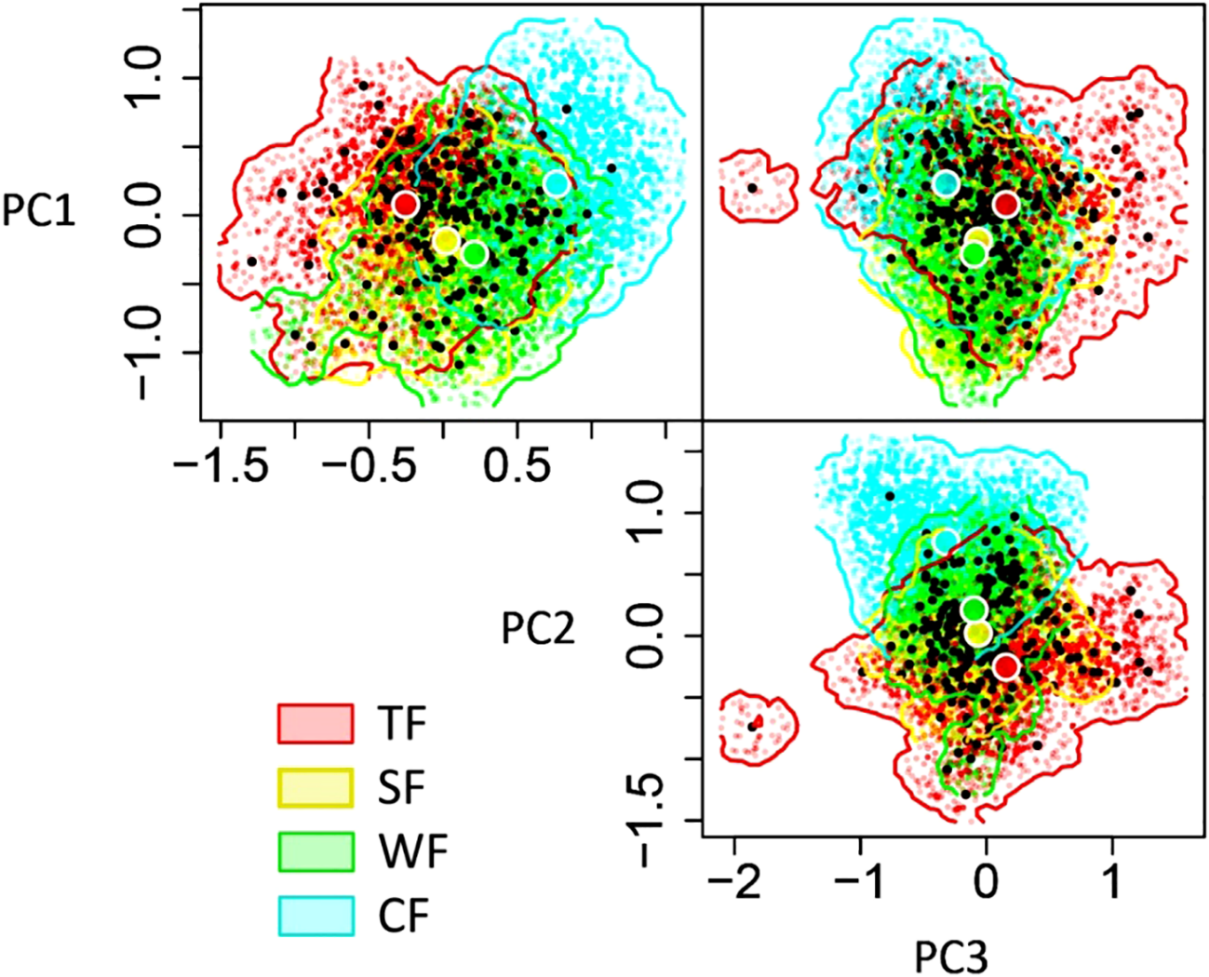
Differences in functional niche hypervolumes among woody plants in various forest vegetation types. The three dimensions of N-dimension hypervolumes are defined based on PC1-PC3, where PC1-PC3 presents the first three principal component axes of functional niche space for woody plants.TF, Tropical rainforest; SF, Subtropical evergreen deciduous broad-leaved mixed forest; WF, Warm-temperate coniferous broad-leaved mixed forest; CF, Cold-temperate coniferous forest.

The functional niche hypervolume of woody forest vegetation decreases from tropical to cold temperate zones. The largest hypervolume is observed in the TF (niche hypervolume = 4.75), followed by coniferous woody plants in CF (niche hypervolume = 3.59), while SF (niche hypervolume = 2.31) and WF (niche hypervolume = 2.42) exhibit similar values. Notably, the functional niche hypervolume of the woody plants in the TF is significantly larger than the other three forest types: 2.06 times that of the SF, 1.96 times that of WF, and 1.3 times that of CF (**Figure 2**).

**Figure 3:**
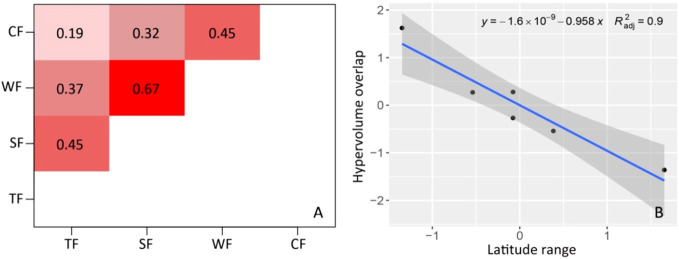
Overlaps of functional niche hypervolumes and their differences among woody plants in different forest vegetation types. **(A)** Overlap index of functional niche; **(B)** Relationship between hypervolume overlap of functional niche and latitude range (*P*<0.001). TF, Tropical rainforest; SF, Subtropical evergreen-deciduous broad-leaved mixed forest; WF, Warm-temperate coniferous broad-leaved mixed forest; CF, Cold-temperate coniferous forest.

The degrees of functional niche overlap among woody plants in different forest vegetation types illustrate the law of geographical differentiation (**Figure 3**). The degree of overlap significantly decreases with increasing latitude span, indicating an increase in functional differences. There is a notable disparity in the degree of functional niche overlap among different forest vegetation types (**Figure 3**). The overlap degree for TF and CF plants is the lowest (overlap index = 0.19), while that of SF and WF plants is the highest (overlap index = 0.67). The degree of overlap between TF and SF is 0.45, that of WF is 0.37, and that of CF is 0.19. The degree of overlap between SF, WF, and CF are 0.67 and 0.32, respectively. The degree of overlap between WF and CF is 0.45.

### 3.2 Relationship between functional niche hypervolume of forest vegetation and climate and soil factors

The combined impact of climate and soil environmental factors accounts for 50% of the total volume differentiation in the functional niche of woody plants across different forest vegetation types. Within the variance explained by environmental factors, the joint explanatory power of climate and soil factors on the variation in functional niche is 6%. Independently, climate factors contribute 28% to the explanation, while soil factors independently contribute 16%. This implies that the explanatory power of climate factors is 12% higher than that of soil factors. These findings suggest that climate factors exert a greater influence on the variation in functional niche hypervolume among forest vegetation types than soil factors do.

**Figure 4:**
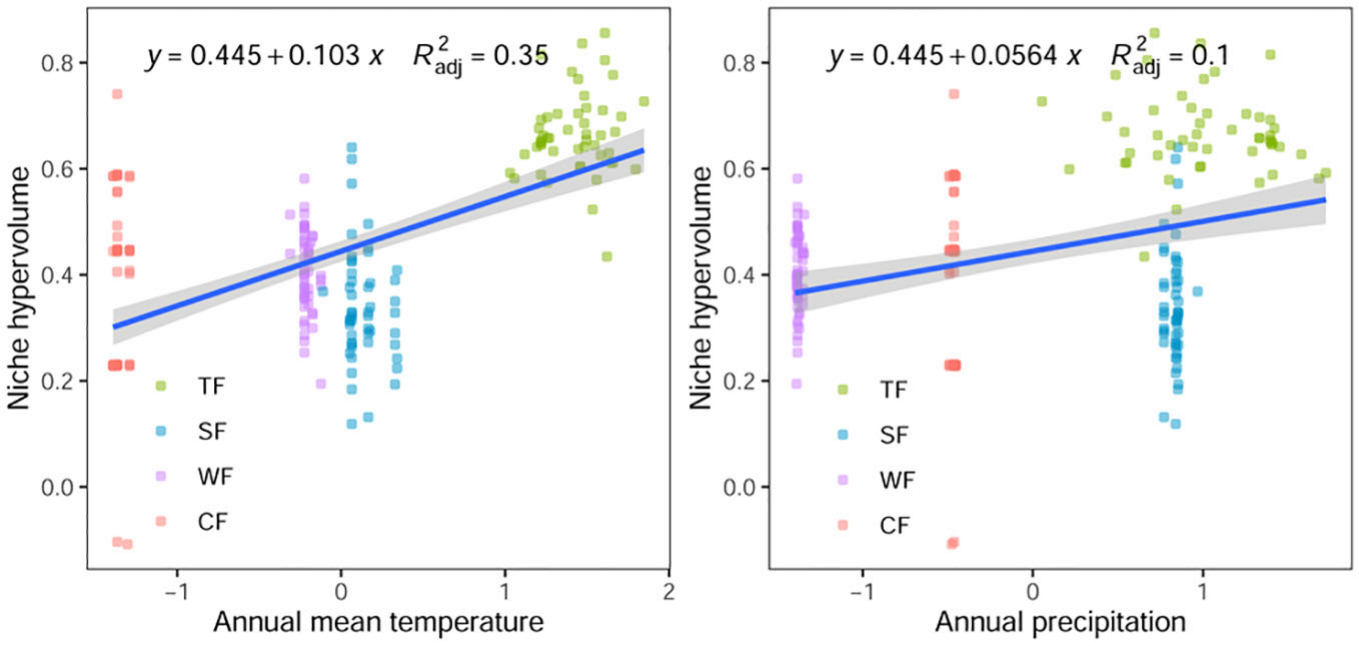
Relationships between climatic factors and functional niche hypervolumes of woody plants in different forest vegetation types. All *P* values are less than 0.01. TF, Tropical rainforest; SF, Subtropical evergreen deciduous broad-leaved mixed forest; WF, Warm-temperate coniferous broad-leaved mixed forest; CF, Cold temperate coniferous forest.

**Figure 5:**
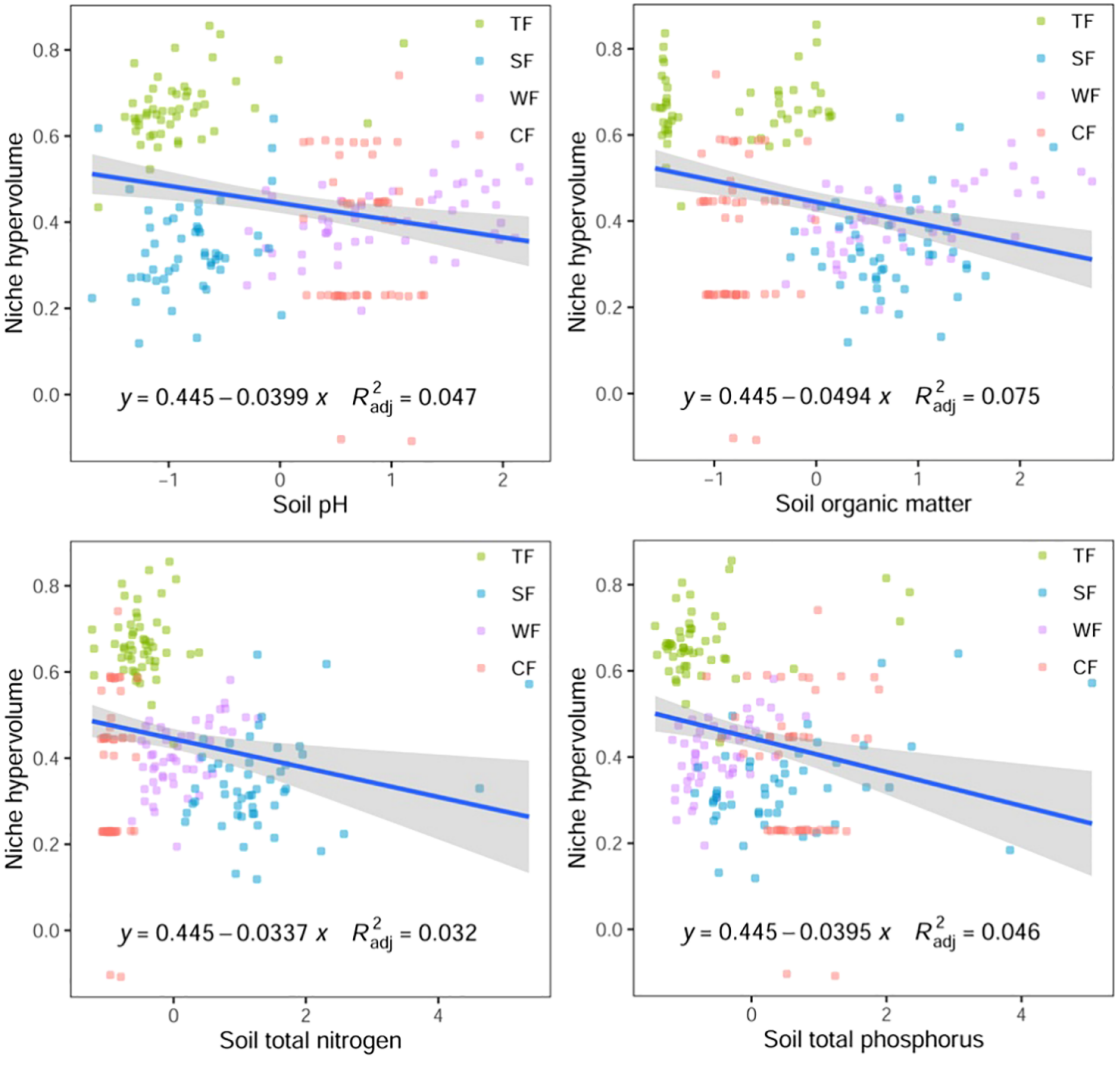
Relationships between soil factors and functional niche hypervolumes of woody plants in different forest vegetation types. All P values are less than 0.01. TF, Tropical rainforest; SF, Subtropical evergreen deciduous broad-leaved mixed forest; WF, Warm-temperate coniferous broad-leaved mixed forest; CF, Cold temperate coniferous forest.

The results of Pearson correlation analysis reveal a significant correlation between the functional niche hypervolume of woody plants in different forest vegetation and six environmental factors, excluding the contents of soil available nitrogen, phosphorus, and potassium (P < 0.01, **Table 1**). Regression analysis further indicates a noticeable trend in the difference of functional niche hypervolume concerning several environmental factors. The functional niche hypervolume demonstrates a positive correlation with the annual average temperature and annual precipitation. Woody plant communities experiencing higher temperatures and precipitation exhibit larger functional niche hypervolume measurements (**Figure 4**). Additionally, the functional niche hypervolume shows a negative correlation with the four soil factors. Woody plant communities with higher values of soil pH, as well as content of organic matter, nitrogen, and phosphorus, exhibit smaller functional niche hypervolume measurements (**Figure 5**).

**Table 1:** Correlations between environmental factors and the functional niche hypervolume of woody plants in different forest vegetation types.

	NH	AMT	APRE	pH	SOM	TN	TP	AN	AP	AK
NH	1									
AMT	0.591^***^	1								
APRE	0.323^***^	0.605^***^	1							
pH	-0.228^**^	-0.656^***^	-0.826^***^	1						
SOM	-0.283^***^	-0.073	-0.228^**^	0.201^**^	1					
TN	-0.193^**^	0.171^*^	0.223^**^	-0.217^**^	0.708^***^	1				
TP	-0.226^**^	-0.433^***^	0.036	0.18^*^	0.188^**^	0.35^***^	1			
AN	-0.038	0.364^***^	0.171^*^	-0.208^**^	0.72^***^	0.884^***^	0.166^*^	1		
AP	-0.174^*^	-0.622^***^	-0.263^***^	0.438^***^	-0.367^***^	-0.513^***^	0.366^***^	-0.617^***^	1	
AK	-0.142^*^	-0.458^***^	-0.374^***^	0.559^***^	0.01	-0.059	0.41^***^	-0.091	0.538^***^	1

^***^, ^**^, and ^*^ indicate *P*<0.001, *P*<0.01, and *P*<0.05, respectively. NH, Niche hypervolume; AMT, Annual mean temperature; APRE, Annual precipitation; pH, Soil pH value; SOM, Soil organic matter; TN, Soil total nitrogen; TP, Soil total phosphorus; AN, Soil available nitrogen; AP, Soil available phosphorus; AK, Soil available calcium.

### 3.3 Key environmental factors affecting the difference in functional niche hypervolumes of forest vegetation

The influence of environmental factors on the difference in functional niche hypervolume of forest vegetation varies by variable (**Table 2**). The difference significantly responds to changes in annual average temperature (P < 0.001) and is even more pronounced in response to changes in soil pH, soil total nitrogen, and total phosphorus contents (P < 0.01). According to the results of variable importance inferred from the MSMI multi-model, annual average temperature and soil pH emerge as the most crucial variables determining the difference in functional niche hypervolume of forest vegetation, each with a variable importance value of 1.00. The importance of soil total nitrogen content is somewhat weaker, with a variable importance value of 0.99, followed by the soil total phosphorus content, which has a variable importance value of 0.98. These results underscore that annual average temperature, soil pH value, and soil total nitrogen content are the key environmental factors influencing the difference in functional niche hypervolume of forest vegetation.

**Table 2:** Importance values of environmental factors on functional niche hypervolumes of forest vegetation.

Variables	Z value	*P* value	Variable importance
Intercept	50.943	<0.001	
AMT	10.759	<0.001	1.00
pH	3.197	<0.01	1.00
TN	3.685	<0.01	0.99
TP	2.902	<0.01	0.98
APRE	0.801	0.423	0.55
SOM	0.540	0.589	0.41

AMT, Annual mean temperature; pH, Soil pH value; TN, Soil total nitrogen; TP, Soil total phosphorus; APRE, Annual precipitation; SOM, Soil organic matter.

## 4 Discussion

### 4.1 The rule of difference in functional niche hypervolumes among forest vegetation types

The functional niche hypervolume represents the spatial extent of the functional niche occupied by a species, indicating the richness of the functional diversity within a community. Larger functional niche hypervolumes in communities correspond to higher species functional diversity. In our study, the functional niche hypervolume of woody plant communities across different climatic regions exhibited a decreasing trend from south to north. The tropical rainforest, with the largest functional niche space among the four study areas, likely possessed the highest functional diversity. This elevated functional diversity was associated with superior water and heat conditions, fostering high species richness. Research has shown that the functional niche space increases with species richness due to greater functional differences (Swenson and Weiser, 2014), emphasizing the complementary effect of enhanced plant functional niches with increasing functional niche space. Consequently, differences in functional diversity between communities are largely attributed to variations in species number and composition.

Interestingly, the SF has a slightly higher number of species than that of the WF. However, the functional niche hypervolume of the woody plants in the SF is slightly smaller than that in the temperate forest. This may suggest a more concentrated functional niche in the subtropical region compared to the temperate region. The spatial convergence of the functional niche of species at lower latitudes may be stronger than that at higher latitudes. In our study, the woody plants in the subtropical region are all broad-leaved trees, while temperate forests include both broad-leaved and coniferous trees. Therefore, the leaf function characteristics of woody plants in subtropical forests are similar, while those in temperate forests are more differentiated in the leaf economic dimension. Additionally, the SF has more abundant water and heat resources than the WF, indicating more intense competition for woody plant resources in the temperate forest. The expansion of the functional niche space of woody plants in the temperate forest facilitates more effective utilization of environmental resources. The CF has only six coniferous species, but they exhibit large functional niche hypervolumes. This is because the functional differentiation among these few species is highly diverse despite the simplicity of species composition. For example, the leaf area of *Picea obovata* is only 0.14 cm^2^, while the leaf area of *Betula pendula* is 96 times larger.

It is crucial to note that the selection of functional traits can influence our results. The calculation of the functional niche hypervolume is based on the chosen functional traits, and different trait selections may yield different effects on the functional niche hypervolume. While we aimed to avoid subjective trait selection, the traits chosen are limited by the current data availability. We incorporated as many relevant traits as possible for analysis, but not all coefficients were included in this study. Therefore, our results serve as a preliminary exploration, and further analysis with more comprehensive trait data is necessary for more convincing conclusions. Additionally, not all of the traits obtained come from first-hand field investigations; we integrated literature data with field investigation data for the analysis. As the quantity and accuracy of trait data improve, future studies on functional niches based on these data may yield more meaningful results.

### 4.2 The impact of environmental factors on the difference in functional niche hypervolume among forest vegetation types

The influence of various environmental factors on community ecological processes manifests in diverse ways, underscoring the need for a comprehensive consideration of these factors within the sample plot. In this study, the combined impact of two key environmental factors, climate and soil, strongly explains the differentiation in functional niche hypervolume among woody plants in distinct climate regions, accounting for a significant 50% of the variance change. This underscores the close relationship between the functional niche hypervolume of woody plant communities and their environment. Different environments drive changes in geographical patterns of plant functional traits, leading to a geographical differentiation of measures of plant functional niche based on these traits (Reich and Oleksyn, 2004). The four woody plant communities studied here span the tropical, subtropical, warm temperate, and cold temperate zones from south to north, respectively. The variations in the spatial distribution of temperature, precipitation, soil, and other resources significantly impact the functional niche hypervolume across different climatic regions (Duivenvoorden and Cuello A, 2012). Biogeographically, climate emerges as a more dominant factor than soil and terrain in differentiating plant functional traits across regions. Consequently, climate filtering plays a pivotal role in shaping species distribution and functional diversity (Van Nuland et al., 2020; Yao et al., 2020). We observed that the climate factors had greater explanatory power than soil factors in influencing the differences in functional niche hypervolume of woody plant communities. This suggests that climate heterogeneity was the primary environmental factor driving changes in the functional diversity of diverse forest plants. This observation aligns with the “environment energy hypothesis” based on climate (Brown, 2014), showing that energy serves as the driving force behind biodiversity changes. Solar radiation contributes to heat energy, determines factors like average annual temperature and evapotranspiration, and regulates the physiological activities of plants. Consequently, in environments with varying levels of energy, plants tend to exhibit greater functional differentiation to efficiently obtain survival resources (Tilman et al., 2001). Thus, the amount of energy in different vegetation communities plays a crucial role in shaping the functional niche hypervolume of plants.

### 4.3 Key environmental factors influencing the differences in functional niche hypervolume among forest vegetation types

Many studies have shown that there are corresponding laws of change in plant spatial distribution and environmental gradients. The impact of climate heterogeneity on species distribution and diversity at different latitudes has received particular attention (Wilson et al., 2013; Dias et al., 2020; Liu et al., 2022). In general, biodiversity declines with increasing latitude, temperature, precipitation, and evaporation rates (Gaston, 2000). The functional niche hypervolume of woody plant communities across different climate regions also exhibits an obvious pattern of difference. Functional niche hypervolume increases with decreasing latitude and increasing temperature and precipitation. Increased functional niche space corresponds to higher functional diversity, indicating that the trend of functional diversity and species diversity change is mostly consistent at a large scale (Swenson and Weiser, 2014; Li et al., 2018). It is reported that high functional diversity is related to more heterogeneous climate conditions because such conditions enable species to use more resources, allowing a greater number of species to coexist (Stark et al., 2017).

This study found a positive correlation between the functional niche hypervolume of woody plant communities and hydrothermal conditions, which aligns with the environment energy hypothesis. It is recognized in the literature that energy-rich communities can accommodate a greater number of species, thus occupying a larger niche space. The functional niche hypervolume of woody plant communities may also be related to climate stability. Research has shown that areas with stable climates can provide a steady supply of resources necessary for species’ survival, making them more conducive to species’ adaptation and evolution (Jetz et al., 2004; Feng et al., 2019). Tropical rainforests, with the smallest degree of seasonal change and the most stable climate, exhibit the largest functional niche hypervolume of woody plant communities.

This study also identified a negative correlation between functional niche hypervolume of woody plant communities across different climate regions and soil pH value, aligning with previous research (Ruiz-Benito et al., 2017; Šímová et al., 2019). One possible explanation is that soils with higher pH values may reduce the efficiency of plant photosynthetic utilization, consequently limiting plant functional changes and functional niche space occupied by species.

Environmental factors vary in their importance to the functional niche hypervolume of woody plant communities. The findings of this study highlight that annual average temperature is the primary factor influencing the differences in functional niche hypervolume, followed by soil pH and soil total nitrogen content. This underscores the greater significance of climate factors compared to soil factors at large scales. Given the ongoing rapid changes in the global environment, including frequent extreme temperature and precipitation events, it is crucial to comprehend how climate factors affect the functional niche space of species for effective biodiversity conservation planning and implementation. Examining plant functional niches through the lens of functional traits enables scholars and decision-makers to gain a deeper understanding of how these traits shape plant distribution, potentially enhancing species resilience in the face of climate change (Treurnicht et al., 2020). The results of this study suggest that future ecological research should particularly focus on the potential impacts of temperature changes, especially in the context of global climate shifts, on functional niches and biodiversity.

## 5 Conclusions

The novelty of our study lies in revealing the differences in functional niche hypervolume and degree of overlap among woody plant communities across four distinct climate regions. (1) The functional niche hypervolume decreases with increasing latitude, transitioning from tropical rainforest to cold temperate coniferous forest. (2) The degree of functional niche overlap gradually decreases with increasing latitude. (3) The explanatory power of climate and soil factors on the functional niche hypervolume of woody plant communities across different climatic regions was 50%, with the former showing a higher influence than the latter. This suggests that climate is a more critical factor than soil in determining functional niche hypervolume at a large scale. (4) The key climate and soil factors impacting functional niche hypervolume include annual average temperature, soil pH value, and soil total nitrogen content. The study provides a scientific reference for future research exploring the mechanisms of plant community construction from the perspective of functional niche.

## Data availability statement

The raw data supporting the conclusions of this article will be made available by the authors, without undue reservation.

## Author contributions

RZ and JH designed this research project. RY and JH finished the field work. JH and RY analyzed the data and wrote the manuscript. All authors contributed to the article and approved the submitted version.
